# Molecular Hydrogen Is Involved in Phytohormone Signaling and Stress Responses in Plants

**DOI:** 10.1371/journal.pone.0071038

**Published:** 2013-08-12

**Authors:** Jiqing Zeng, Mingyong Zhang, Xuejun Sun

**Affiliations:** 1 Key Laboratory of South China Agricultural Plant Genetics and Breeding, Key Laboratory of Plant Resources Conservation and Sustainable Utilization, South China Botanical Garden, Chinese Academy of Sciences, Guangzhou, China; 2 Department of Diving Medicine, Second Military Medical University, Shanghai, China; Ben-Gurion University of the Negev, Israel

## Abstract

Molecular hydrogen (H_2_) metabolism in bacteria and algae has been well studied from an industrial perspective because H_2_ is viewed as a potential future energy source. A number of clinical trials have recently reported that H_2_ is a therapeutic antioxidant and signaling molecule. Although H_2_ metabolism in higher plants was reported in some early studies, its biological effects remain unclear. In this report, the biological effects of H_2_ and its involvement in plant hormone signaling pathways and stress responses were determined. Antioxidant enzyme activity was found to be increased and the transcription of corresponding genes altered when the effects of H_2_ on the germination of mung bean seeds treated with phytohormones was investigated. In addition, upregulation of several phytohormone receptor genes and genes that encode a few key factors involved in plant signaling pathways was detected in rice seedlings treated with HW. The transcription of putative rice hydrogenase genes, hydrogenase activity, and endogenous H_2_ production were also determined. H_2_ production was found to be induced by abscisic acid, ethylene, and jasmonate acid, salt, and drought stress and was consistent with hydrogenase activity and the expression of putative hydrogenase genes in rice seedlings. Together, these results suggest that H_2_ may have an effect on rice stress tolerance by modulating the output of hormone signaling pathways.

## Introduction

Hydrogen is the lightest and most abundant chemical element in the universe, constituting about 75% of its elemental mass [1]. Hydrogen gas (H_2_) is colorless, odorless, and tasteless, and it has been known as a reducing agent since 1671 when it was first produced by Robert Boyle [1]. However, H_2_ is rare on earth, constituting less than 1 part per million of the atmosphere, and the majority of hydrogen atoms are found in water and organic compounds [2]; nonetheless, it is believed to have been highly concentrated in the atmosphere of the early Earth [3]. H_2_ is considered to be a physiologically inert gas, and it is generally regarded as a potential future energy source and an alternative to limited fossil fuel resources [4].

H_2_ has recently received worldwide attention due to its potential exploitation as a therapeutic medical gas. In 1975, Dole *et al*. first reported significant tumor regression in patients with squamous cell carcinoma who were exposed to hyperbaric H_2_ for 14 days [5]. After 25 years, another study reported that hyperbaric H_2_ could alleviate schistosomiasis-associated chronic liver inflammation [6]. In 2007, Ohsawa *et al.* reported that H_2_ could selectively reduce the hydroxyl radical (**^•^**OH) and peroxynitrite (ONOO^−^), but it does not affect physiological reactive oxygen species (ROS). They suggested that this antioxidant activity could have preventive and therapeutic applications [7]. These reports greatly changed the stereotype of H_2_ and attracted worldwide attention. More than 200 papers describing therapeutic uses of H_2_ have been published since then. The accumulated evidence from a variety of biomedical fields and clinical and experimental models of many diseases indicate that H_2_ administered by gas inhalation or by oral ingestion of H_2_-saturated water acts as a therapeutic agent [8]. These studies have targeted a diverse range of disorders and organ systems including the nervous, digestive, cardiovascular, and respiratory systems [8]. The effects of H_2_ on various diseases have been documented for 63 disease models and human diseases in recent years [9]. The mechanism of hydrogen’s effects in various diseases has mainly been attributed to specific scavenging activity for **^•^**OH and ONOO^−^ [7], [9], [10], and only a few reports have focused on its gene regulation [11] and signal-modulating activities [9], [12], [13].

It has long been known that microbes ranging from ancient bacteria to eukaryotic microalgae are capable of producing hydrogen through photosynthetic and nonphotosynthetic processes [14], [15], [16]. H_2_ metabolism in bacteria and algae has been well studied [17], [18]. However, only a few studies have reported H_2_ metabolism in higher plants. In 1947, Boichenko studied H_2_ evolution in isolated chloroplasts and postulated the existence of hydrogenase in some higher plants [19]. Although it is well known that cyanobacteria and eukaryotic microalgae can photobiologically produce H_2_ using either hydrogenase or nitrogenase [20], [21], whether hydrogenase is present in higher plants or not remains unclear. Based on the fact that hydrogenase is found in many different microorganisms and algae as well as on the presumption of its important role in the reducing environment of the atmosphere of the primitive Earth, Renwick *et al.* (1964) suggested that H_2_ metabolism was possible in many higher organisms [22]. However, no reports have been published to support the above deduction in plants. Here, we present data to support the physiological functions of H_2_ in plants, its interactions with the metabolism and signaling of plant hormones, its production in rice seedlings, and its involvement in response to abiotic stress.

## Results

### Biological Effects of H_2_ on Higher Plants

**Figure 1:**
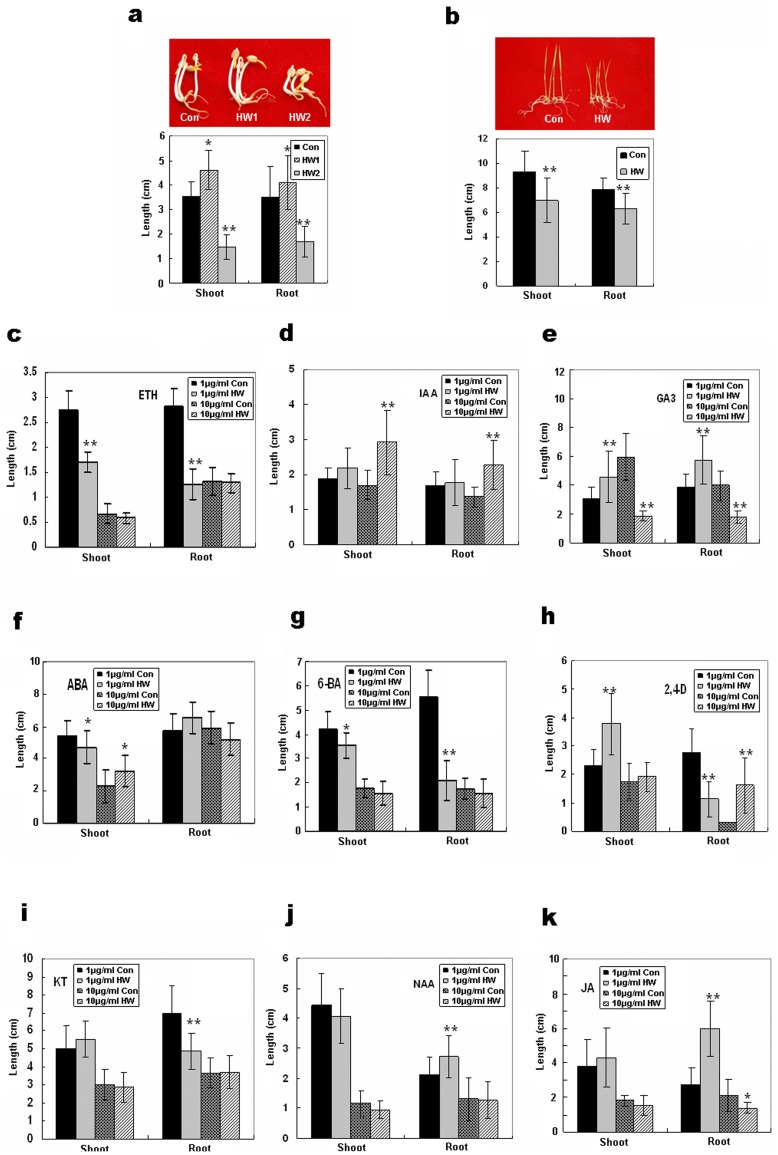
Effects of Hydrogen on Plants. **a**, Germination of mung bean seeds in hydrogen-rich water (HW1∶100 µmol/L; HW2∶350 µmol/L) and in de-hydrogen water (DW) (Control) for 3 d. **b,** Germination of rice seeds in water and HW (250 µmol/L) for 2 weeks. **c–k**, Germination of mung bean seeds in Ethephon (ETH) (**c**), indolylacetic acid (IAA) (**d**), gibberellic Acid-3 (GA_3_) (**e**), abscisic acid (ABA) (**f**), 6-benzylaminopurine (6-BA) (**g**), 2,4-dichlorophenoxyacetic acid (2,4-D) (**h**), kinetin (KT) (**i**), 1-naphthaleneacetic acid (NAA) (**j**), and Jasmonic acid (JA) (**k**) solutions (1 µg/ml and 10 µg/ml) made up with DW (Control) and HW (250 µmol/L). Lengths of shoots and roots of mung bean and rice seedlings were measured and is presented here as the mean ± SE (n = 45, *P<0.05, **P<0.01 compared with the control group, *t*-test).

To determine whether hydrogen has an effect on plants, mung beans were soaked in hydrogen-rich water (HW) (HW1∶100 µmol/L; HW2∶350 µmol/L) and germinated for 3d in darkness. Rice seeds were also germinated in HW (250 µmol/L) in a growth chamber for 2 weeks. Lengths of shoots and roots were then measured respectively. To our surprise, we found a remarkable difference between HW-treated and control plants (Figure 1a, b). The growth of mung bean shoots and roots was accelerated by a relatively low concentration of HW and was suppressed by a relatively high concentration of HW (Figure 1a). The growth of rice seedlings was inhibited by HW (Figure 1b). These results suggested that H_2_ may have biological effects on plants.

Further experiments revealed that H_2_ enhanced the effects of ethylene when mung bean seeds were soaked in low concentrations of ethephon (ETH) (Figure 1c). Ethephon is the most widely used plant growth regulator, which can be converted very quickly to ethylene. Therefore, to test whether H_2_ would also affect the effects of other plant hormones on plant growth and development, mung bean seeds were soaked in relatively low (1 µg/ml) and high concentrations (10 µg/ml) in the presence or absence of HW (250 µmol/L), and H_2_ was found to alter the effect of individual plant hormones (Figure 1d–k). For example, HW significantly prevented the growth inhibition of a high concentration solution of indolylacetic acid (IAA) (Figure 1d). In addition, compared with controls, H_2_ accelerated the growth of mung beans in the presence of a low concentration solution of gibberellic acid (GA_3_) but significantly inhibited growth at higher concentrations of GA_3_ (Figure 1e). Similarly, HW altered the outcome of jasmonic acid (JA), abscisic acid (ABA), 6-benzylaminopurine (6-BA), 2,4-dichlorophenoxyacetic acid (2,4-D), kinetin (KT), and 1-naphthaleneacetic acid (NAA) treatments (Figures 1k, f, g, h, i, j). These results show that H_2_ appears to have opposite effects on different concentrations of these plant hormones, which indicates that H_2_ may have biological effects on plants due to synergistic or antagonistic interactions with plant hormones.

### Plant Hormone Receptor Gene Transcription was Regulated by HW

**Figure 2:**
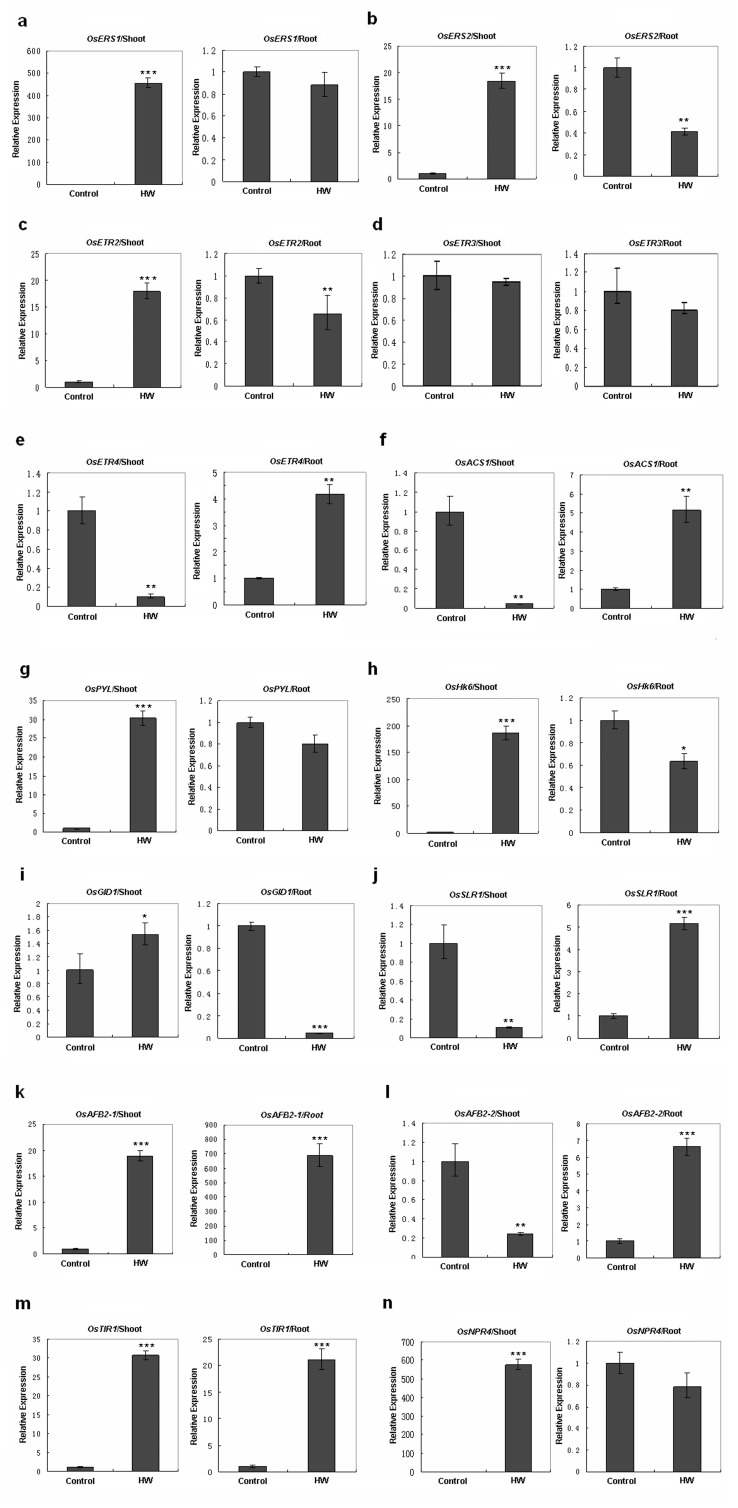
Expression of genes involved in the plant hormone signaling pathway in rice seedling shoots and roots regulated by H_2_. Rice seeds were germinated and maintained in hydrogen-rich water (HW) or de-hydrogen water (Control) for 2 weeks. RT-qPCR was performed to estimate the transcription rate of hormone receptor genes and of a few key factors involved in related signaling pathways (**a–e**, ETH receptor genes, *OsERS1, OsERS2*, *OsETR2*, *OsETR3*, and *OsETR4*. **f**, ACC synthetase gene, *OsACS1.* **g**, ABA receptor gene, *OsPYL*. **h**, CTK receptor gene, *OsHk6*. **I**, GA receptor gene, *OsGID1*. **j**, a gene for a key transcript factor (DELLA protein OsSLR1) in the GA signaling pathway. **k–m**, IAA receptor genes, *OsAFB* and *OsTIR*. **n**: SA receptor gene, *OsNPR4*. Data are presented as mean ± SE, n = 3, *P<0.05, **P<0.01, ***P<0.001 compared with the control group (*t*-test).

To confirm the above observations, expression of receptor genes for several plant hormones was investigated in rice. Rice seeds were germinated and grown in glass dishes with or without HW (250 µmol/L) for 2 weeks (Figure 1b). Reverse-transcription quantitative PCR **(**RT-qPCR) was performed to quantify the expression of these genes in rice seedlings. A total of 12 genes for phytohormone receptors were analyzed, including 5 ethylene receptor genes (*OsERS1*, *OsERS2*, *OsETR2*, *OsETR3*, and *OsETR4*) [23], 3 auxin (IAA) receptor genes (*OsAFB2-1*, *OsAFB2-2*, *OsTIR1*) [24], [25], 1 abscisic acid (ABA) receptor gene (*OsPYL*) [26], 1 gibberellin (GA) receptor gene (*OsGID1*) [27], 1 cytokinin (CTK) receptor gene (*OsHk6*) [28], and 1 salicylic acid (SA) receptor gene (*OsNPR4*) [29]. In addition, an ACC synthase gene (*OsACS1*), the gene for a key transcript factor (*OsSLR1*) in the GA pathway and a gene related to pathogenesis (*OsPR1*) in the SA signaling pathway were also investigated. Expression of most of these genes dramatically changed in the shoots or roots of rice seedlings treated with HW (Figure 2, Table S1). In particular, changes in the transcription of plant hormone receptor genes were greater in the shoots than in the roots (Figure 2). As roots were soaked in HW, the concentration of H_2_ should be higher in the roots than in the shoots. These data suggest that low H_2_ concentrations may enhance the expression of plant hormone-related genes, whereas higher concentrations may suppress expression. An alternative or complementary explanation is that the shoots of rice seedlings may be more sensitive to H_2_ than the roots.

### Rice Seedlings can Generate H_2_, and its Production is Regulated by Hormones and Stress

**Figure 3:**
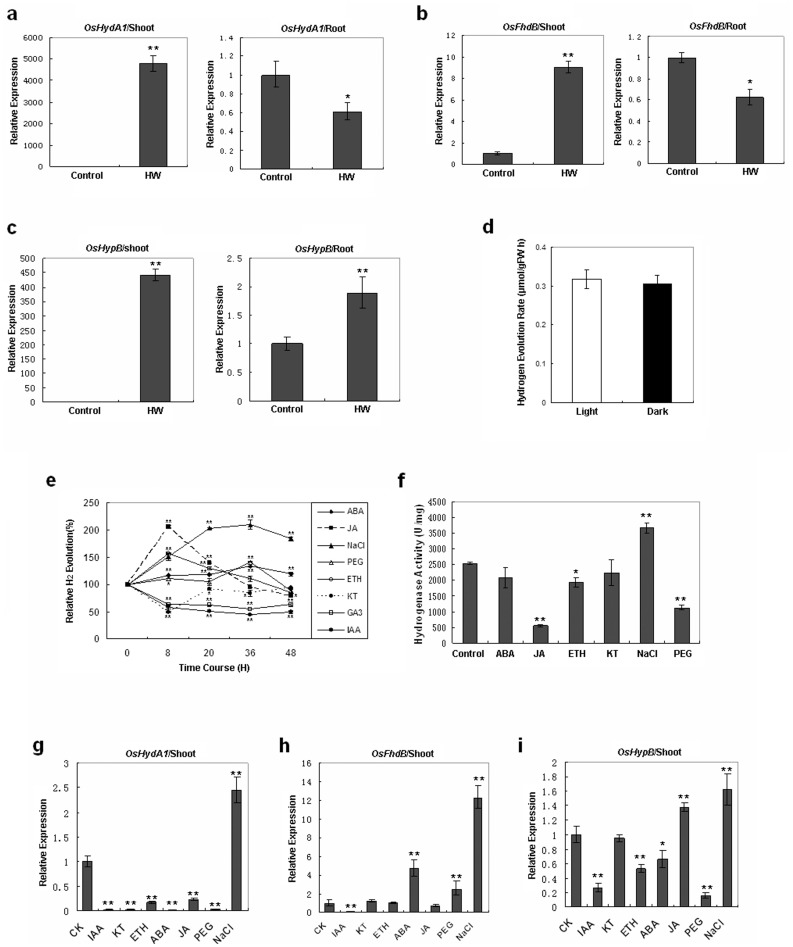
Endogenous production of hydrogen by rice seedlings. **a–c,** Expression of putative rice hydrogense genes, *OsHydA1*, *OsFhdB*, and *OsHypB*, in rice seedlings. Rice seeds were germinated and maintained in hydrogen-rich water (HW) or de-hydrogen water (Control) for 2 weeks. RT-qPCR was performed to investigate the expression of these genes in shoots and roots. **d**, Hydrogen evolution rate in rice seedlings stored under light and dark conditions. **e**, Relative H_2_ evolution in rice seedlings treated with ABA (1 µg/ml), JA (1 µg/ml), ETH (1 µg/ml), KT (1 µg/ml), GA_3_ (1 µg/ml), IAA (1 µg/ml), PEG6000 (5%), and NaCl (1%). **f**, Hydrogenase activity of the treated rice seedlings after 2 d. **g–i**, Expression of putative rice hydrogense genes, *OsHydA1*, *OsFhdB*, *OsHypB*, evaluated by RT-qPCR in rice seedling shoots after 2-day treatment with various hormones and exposure to different stresses. Data are presented as mean ± SE, n = 3, *P<0.05, **P<0.01 compared with the control group (*t*-test).

Considering the great effects of H_2_ on plants described above, we aimed to determine whether H_2_ could be produced endogenously in higher plants. To the best of our knowledge, biological H_2_ production is primarily found in microbial species [30]. However, there are at least three putative hydrogenase gene homologs in rice: *OsHydA1* (AK067853, Os03g0748700), *OsFhdB* (AK068015, Os04g0320100), and *OsHypB* (AK110758, Os01g0309800) (Figures S1, S2, S3). We performed an RT-qPCR to test whether the expression of these genes could be altered by H_2_. We found that transcription of the three putative rice hydrogenase genes was dramatically upregulated by 2-week exposure to HW (Figure 3a–c).

We analyzed the gas collected in a glass beaker (250 ml) covering rice seedlings in a growth chamber using a H_2_ detector to determine whether rice seedlings could produce H_2_. Concentration of H_2_ in the glass beaker was measured every 2 h. After measurement, the glass beaker was opened to replace the seedling headspace with fresh air. At the end of the experiment, the seedlings were weighed and the evolution of H_2_ was estimated to be 0.318 µmol gFW^−1^ h^−1^ under light and 0.305 µmol gFW^−1^ h^−1^ in the dark (Figure 3d, Table S2). To further characterize H_2_ production in rice, the production of H_2_ was analyzed in rice seedlings treated with plant hormones or under abiotic stresses. Before treatment, the concentration of H_2_ in a glass beaker covering dishes of rice seedlings was quantified every 2 h to make sure that the evolution of H_2_ was stable. Relative H_2_ evolution was defined as the ratio of H_2_ concentration after treatment to the average H_2_ concentration before treatment (data are shown in Table S3). H_2_ production increased rapidly in the rice seedlings treated with ETH (1 µg/ml), JA (1 µg/ml), ABA (1 µg/ml), 5% PEG6000, and 1% NaCl; in contrast, H_2_ production was decreased in seedlings treated with KT (1 µg/ml), GA_3_ (1 µg/ml), and IAA (1 µg/ml). The increased H_2_ production was maintained for 36 h in the groups treated with ABA, NaCl, and PEG6000 but decreased after 8 h in the groups treated with ETH and JA (Figure 3e). A rapid reduction in H_2_ production was also observed in rice seedlings soaked in a 855 mM NaCl solution (data not shown). These data demonstrated that H_2_ could be endogenously produced by plants and that this production could be affected by plant hormones and abiotic stresses.

Hydrogenase activity in rice seedlings was measured after treatment with various plant hormones and stresses for 2 d to examine whether H_2_ was generated by hydrogenase enzymes in rice. The hydrogenase activity in rice seedling shoots treated with 171 mM NaCl for 2 d was higher than that of the control shoots, whereas the hydrogenase activity of rice seedling shoots under the other experimental conditions was lower than that of the controls (Figure 3f). These data are consistent with the different hydrogenase activity values of rice seedlings observed under the various experimental conditions. To confirm the above findings, the expression of putative hydrogenase genes (*OsHydA1*, *OsFhdB*, and *OsHypB*) in rice seedlings was investigated under various experimental conditions. Expression of the three putative rice hydrogenase genes in rice seedling shoots was greatly upregulated by 171 mM NaCl treatment. *OsFhdB* and *OsHypB* expression was upregulated by ABA, 5% PEG6000, and JA, respectively, but was downregulated by IAA (Figure 3g–i). These data show that under NaCl treatment, H_2_ production, hydrogenase activity and mRNA levels of the three putative rice hydrogenase genes are consistent, while under other treatments, they are not always consistent with the three putative rice hydrogenase genes; however, H_2_ production and hydrogenase activity is consistent. Because the hydrogenase activity of rice seedlings is the total enzyme activity of the three putative rice hydrogenase, the different expression profiles of the three putative rice hydrogenase genes suggested that the regulation of these three genes may be different. The production of H_2_ was consistnent with the changes in hydrogenase activity and mRNA levels of putative rice hydrogenase genes under the NaCl treatment strongly suggesting that H_2_ is generated by hydrogenase enzymes in rice seedlings.

### Genes for Antioxidant Enzymes in Rice Seedlings are Upregulated by HW

**Figure 4:**
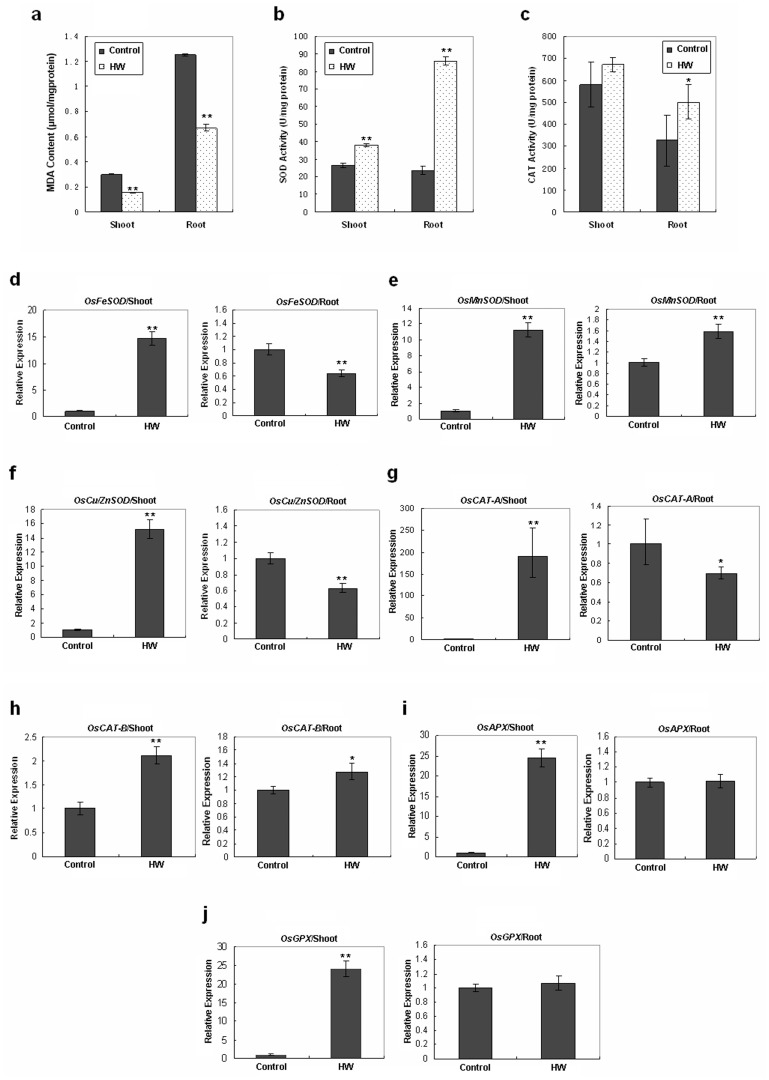
Antioxidant effects of hydrogen in rice seedlings. Rice seeds were germinated and maintained in hydrogen-rich water (HW) for 2 weeks or in de-hydrogen water (DW) as a control. **a,** MDA content in rice seedling shoots and roots. **b**, SOD activity in rice seedling shoots and roots. **c**, CAT activity in rice seedling shoots and roots. **d–j**, Expression of genes for antioxidant enzymes evaluated by RT-qPCR in rice seedling shoots and roots. Data are presented as mean ± SE, n = 3, *P<0.05, **p<0.01 compared with the control group (*t*-test).

Considering the fact that H_2_ is a known therapeutic antioxidant in animal models and humans [9], its antioxidant effects in rice seedlings were also examined. The malondialdehyde (MDA) content of rice seedlings shoots and roots incubated in the presence of HW (250 µmol/L) for 2 weeks was lower than that of control samples (Figure 4a). The activities of antioxidant enzymes such as superoxide dismutase (SOD) and catalase (CAT) were higher in the shoots or roots of the HW-treated seedlings compared with controls (Figure 4b, c). To examine whether the mRNA levels of genes related to antioxidant stress were increased by HW, the transcription of genes for selected antioxidant enzymes (*OsFeSOD*, *OsMnSOD*, *OsCu/ZnSOD*, *OsCAT-A*, *OsCAT-B*, *OsAPX* and *OsGPX*) was investigated by RT-qPCR. We found that all of these genes were upregulated by HW in the shoots or roots (Figure 4d–j). Hepatic oxidoreduction-related genes in rats have been reported to be upregulated by the administration of hydrogen-saturated drinking water [11]. Our results are consistent with this report and indicate that H_2_ may have an antioxidant effect in plants by regulating the expression of genes for antioxidant enzymes, as it does in animal cells.

## Discussion

### The Effect of H_2_ on Higher Plants

Clinical effects of H_2_ was first reported in 1975 [5], but it was not until 2007 that the medicinal value of H_2_ aroused worldwide interest [7]. The effects of H_2_ on animals and humans have mainly been considered to be due to its antioxidant properties and its targeting of cytotoxic oxygen radicals [7], [9], [10]. However, the effects of H_2_ have rarely been studied in higher plants. In this study, we found decreased MDA content in rice seedlings treated with HW (Figure 4a), indicating that H_2_ may act as an antioxidant agent in higher plants as well as in animals. We also analyzed the activities of antioxidant enzymes such as SOD and CAT (Figure 4b, c) as well as the transcription level of genes encoding antioxidant enzymes (*OsFeSOD, OsMnSOD, OsCu/ZnSOD, OsCAT-A, OsCAT-B, OsAPX and OsGPX*) (Figure 4d–j) and found that H_2_ could trigger the expression of these genes and increase the activity of the associated antioxidant enzymes. One animal study has shown that besides acting as a ROS-scavenger or inducer of antioxidant systems, H_2_ may also influence signal transduction and thus act as a novel signaling molecule [6]. A recent study on plants demonstrated that H_2_ functions as a signaling molecule rather than an antioxidant in salt stress resistance [31]. Although the possibility of direct ROS scavenging by H_2_ cannot be totally ruled out in plants, our findings confirm that H_2_ can function as a signaling molecule and regulate the expression of genes for antioxidant enzymes in plants.

In addition to its antioxidant effect, other biological effects of H_2_ on higher plants were found to be different from those in animals and humans in our study. The growth of mung bean and rice seedling roots and shoots was affected by HW pretreatment, suggesting that H_2_ may also have physiological effects on plant growth and development. In our study, 12 genes (including most of the known genes for plant hormone receptors in rice) were found to be upregulated by H_2_, suggesting that H_2_ can interfere with the plant hormone network.

It is well known that plant hormones, such as ethylene, ABA, SA, and JA, are primary signals that regulate protective responses to biotic and abiotic stresses [32], [33]. Extensive studies have shown that plant hormones, such as ethylene, ABA, SA, and JA, can induce an antioxidant defense response against oxidative damage [35]–[41]. In our study, H_2_ pretreatment enhanced the antioxidant capacity of rice seedlings by increasing the expression of antioxidant genes and the activities of antioxidant enzymes, suggesting that H_2_ may be an important signaling molecule in plant responses to stress. A large body of evidence has demonstrated that plant hormones extensively crosstalk to coordinate an appropriate output response to biotic and abiotic stresses [34]. In the present study, H_2_ involvement in the plant hormone signaling pathway was found first, suggesting that H_2_ may trigger the ROS scavenging system by regulating the receptor genes of plant hormones related to the stress response, such as ABA, ethylene, SA, and JA. Therefore, we propose that H_2_ may act as a gaseous bioactive molecule like nitric oxide and ethylene [42].

### H_2_ Involvement in Plant Stress Response may be Due to its Regulation of Plant Hormones

Stress can trigger the production of plant stress hormones such as ABA and ethylene [43], [44]. We similarly found that stress can induce H_2_ production in plants. Although H_2_ metabolism was first reported in 1931 and hydrogenase was first purified from *Clostridium pasteurianum* in 1971, the biological role of H_2_ has not been well studied, and almost all studies of H_2_ metabolism and hydrogenase have focused on bacteria and green algae with the aim of industrial utilization of H_2_ production from cells [17], [22], [45], [46]. H_2_ evolution was observed in higher plants in some early studies [19], [22], but the existence of hydrogenase in higher plants has remained controversial for many years as the possibility of microorganism contamination could not be easily excluded. In 1986, Torres *et al*. demonstrated that H_2_ evolution and hydrogenase activity in barley induced by anaerobic treatment are due to the plant itself and not by contaminant microorganisms [47]. However, they only detected H_2_ evolution and hydrogenase activity in the roots and hypocotyls and not in the leaves. In our study, hydrogenase activity was detected in both the roots and shoots of rice seedlings. Three putative hydrogenase genes in rice, *OsHydA1*, *OsFhdB,* and *OsHypB*, were found to be upregulated in leaves or roots of rice seedlings by HW treatment (Figure 3a–c), suggesting that these three putative hydrogenase genes in rice may have a function in H_2_ metabolism. In addition, we found that production of H_2_ was consistent with changes in the hydrogenase activity and expression of these genes in rice seedlings under NaCl treatment (Figure 3e–i), suggesting that the detected H_2_ evolution and hydrogenase activity in the rice seedling shoots may arise from the plant itself and not contaminant microorganisms. The fact that hydrogenase activity could be detected in the shoots but not the roots of the rice seedlings further suggests that the contribution of contaminant microorganisms, if any, was negligible in our system. We also found that H_2_ evolution was constant and independent of light in rice seedlings grown under normal conditions (Figure 3d), suggesting that a basal physiological level of endogenous H_2_ production is important for normal growth of the plant. It is interesting to note that not only abiotic stress but also treatment with ABA, ETH, and JA (but not IAA, GA_3,_ and KT) induced a rapid increase in the transcription of three putative hydrogenase genes as well as increased H_2_ production (Figure 3e), suggesting that H_2_ may have a role in the plant stress response. In particular, we noticed that expression of the putative rice hydrogenase genes, *OsHydA1*, *OsFhdB*, and *OsHypB*, hydrogenase activity, and H_2_ production were greatly induced in rice seedling shoots exposed to salt stress (Figure 3g–i), which suggests that H_2_ may have an important role in plant salt stress resistance. These results provide strong evidence of H_2_ metabolism in rice and indicate that H_2_ metabolism can be affected by plant hormones and stresses, which itself suggests that H_2_ may act as a signaling molecule in plant adaptation to environmental stress and in response to plant stress hormones, such as ABA, ethylene, NO, SA, and JA.

In conclusion, this is the first report to note the involvement of H_2_ in the plant hormone network and in the stress response in higher plants. H_2_ was found to have multiple roles in higher plants, including being involved in germination and as an antioxidant and signaling molecule in the plant stress response. The increased transcription of phytohormone receptor genes observed in rice seedlings pretreated with HW together with the induction of endogenous H_2_ production by salt and drought stress and by ABA, ETH, and JA suggest that H_2_ may improve rice tolerance to stress via its involvement in the plant hormone signaling pathway. We postulate that H_2_ may be involved in the stress response in higher plants in the form of a gaseous plant hormone like ethylene or NO.

## Materials and Methods

### Generation of Hydrogen-rich Water and De-hydrogen Water

Hydrogen-rich water (HW) was produced according to Nakao *et al*. [48]. De-hydrogen water (DW) was produced by vacuum pumping HW for 30 min. Hydrogen concentration of HW and DW was determined with a “Dissolved hydrogen portable meter” (Trustlex Co., Ltd, ENH-1000, Japan).

### Germination Test of Mung Bean and Rice Seeds

Mung bean seeds were placed in three tissue culture bottles (15 seeds per bottle; in triplicate) and soaked in 50-ml HW. The bottles were covered and wrapped with foil paper to keep the seedlings moist and in darkness for 3 d. DW was used instead of HW in the control bottles. Rice seeds were germinated in a culture dish (45 seeds per dish; in triplicate) for 2 weeks in HW. DW was used instead of HW in the controls. Rice seedlings were cultured in a growth chamber (12 h light-12 h dark cycle, 28°C). After germination, lengths of shoots and roots were measured and counted. All experiments were repeated three times.

To investigate whether hydrogen affects the effects of plant hormones on plant growth and development, mung bean seeds (15 seeds per bottle; in triplicate) were soaked in 5-ml solutions of plant hormones and HW at two concentrations (1 µg/ml and 10 µg/ml) in three culture bottles, as described above. DW was used instead of HW in the controls.

### Reverse-transcription Quantitative PCR (RT-qPCR)

Total RNA was extracted from rice seedling shoots and roots with RNAiso Plus (TAKARA) and treated with DNAse I (Invitrogen) using the standard protocols. Reverse transcription was performed with oligodT primers and a *Promega* reverse transcription kit in accordance with the manufacturer’s instructions. RT-qPCR was performed using SYBR® Premix Ex Taq™ II (TAKARA) on an ABI 7500 machine (Applied Biosystems). Primers used in RT-qPCR are shown in Table S1. Rice Os*Actin1* (LOC_Os03g50885) served as the standard for normalizing the expression of all studied genes. The reactions were incubated at 95°C for 30 s prior to 40 PCR cycles (95°C for 10 s and 61°C for 20 s). All reactions were performed in triplicate. Independent experiments were repeated three times.

### Measurement of H_2_ Production by Rice Seedlings

Rice seeds were germinated for 10 d, with 10 seeds in one culture dish (ΦO = 6.0 cm). Seedlings were treated with various plant hormones (ABA, JA, IAA, GA3, ETH, and KT). Concentration of each plant hormone was identical at 1.0 µg/ml; concentration of PEG600 was 5% and that of NaCl was 1%. When measuring hydrogen production by the rice seedlings, a small dish (ΦO = 6.0 cm) containing the rice seedlings was placed in a bigger dish (ΦO = 9.8 cm) filled with water. The small dish with the rice seedlings was covered with a 250 ml glass beaker with the rubber pipe of the H_2_ gas detector inserted. The opening of the glass beaker was immersed in the water to prevent the inflow of air. Hydrogen production in the glass beaker was detected with a portable H_2_ gas detector (F-7901, China).

### Measurement of Hydrogenase Activity of the Rice Seedlings

Hydrogenase activity of the rice seedlings was measured according to Yu & Wolin (1969) [49] and Peck & Gest (1956) [50] with some modifications. Rice seedling shoots (0.5 g) were homogenized in 100 mM potassium phosphate buffer (pH 7.5) containing 0.1 mM EDTA. The homogenate was then centrifuged at 10000*g* for 10 min at 4°C. The supernatant was then collected to measure the hydrogenase activity, which was performed using a hydrogen reaction system in which methyl viologen (MV) was used as the electron carrier and Na_2_S_2_O_4_ as the electron donor. The hydrogenation reaction system included 30 mmol/L Tris HCl buffer solution (pH = 7.5), 5.25 mmol/L MV, and 1.75 mmol/L Na_2_S_2_O_4_. The total reaction volume was 2 mL. Determination of enzyme activity was performed at 25°C. First, 1 mL of MV solution (5.25 mM in Tris–HCl buffer, 30 mM, pH 7.5) was added in a colorimetric cup. Then 100 µL of the enzymatic extract was added, and the reaction was initiated by adding 900 µL of 1.75 mM Na_2_SO_4_ solution in the same Tris-HCl buffer described above. Extracts active in the spectrophotometric assay were inactivated by heating in a boiling-water bath for 10 min and were used as controls to determine the reduction of MV absorbance in a UV–VIS spectrophotometer (λ = 600 nm). The extinction coefficient was 8.25 mM^−1^×cm^−1^. One unit of enzyme is defined as the quantity of enzyme required to catalyze 1 µmol/L H_2_ every minute [51].

### Assays of Enzyme Activity and Determination of Oxidative Damage

Assays of SOD and CAT activity and the determination of malondialdehyde (MDA) content in rice seedlings were performed according to Jiang and Zhang (2001) [35].

### Statistical Analysis

Data are presented as means ± SE from three independent biological replications. Statistical analyses were performed using SPSS 11.5 software (SPSS Inc.). Differences among treatments were analyzed by paired-sample t test, taking the *P*<0.05 level as a significant and *P*<0.01 as an extremly significant difference.

## Supporting Information

Figure S1 **Amino acid alignment of predicted rice hydrogenase HydA1 protein (Accession No. BAF13185.1) and** ***Chlamydomonas reinhardtii*** **iron hydrogenase HydA1 proteins. Multiple sequence alignment was performed by ClustalX 1.81.** Identical and similar residues are shaded in black and grey, respectively, by ISREC BOXSHADE software (http://www.ch.embnet.org/software/BOX_form.html). Conserved amino acids domains were underlined. Dashes (–) show gaps.
(TIF)

Figure S2 **Amino acid alignment of predicted rice hydrogenase FhdB protein (Accession No. BAF14371.1) and other hydrogenase FhdB proteins (Nostoc sp. PCC 7107 (Accession No. YP_007048442) and** ***Chlamydomonas reinhardtii*** **(Accession No. EDO98609.1)).** Multiple sequence alignment was performed by ClustalX 1.81. Identical and similar residues are shaded in black and grey, respectively, by ISREC BOXSHADE software (http://www.ch.embnet.org/software/BOX_form.html). Dashes (–) show gaps.
(TIF)

Figure S3 **Domain analysis of predicted rice hydrogenase HypB protein (Accession No. BAF04766.1) (A) and other hydrogenase HypB proteins (** ***Methanothermobacter thermautotrophicus str. Delta H*** **(Accession No. NP_275923.1) (B),** ***Methanocella paludicola SANAE*** **(Accession No. YP_003357614.1) (C),** ***Cyanothece sp. PCC 7822*** **(Accession No. YP_003887299.1) (D) and** ***Sphingopyxis alaskensis RB2256*** **(Accession No. YP_611146.1) (E).** The domain analysis was performed at the website of SMART (http://smart.embl-heidelberg.de/), showing that HypB proteins have segments of low compositional complexity (in the color of pink). OsHypB has a histidine-rich region. The histidine residues within the histidine-rich region of HypB are involved in metal binding (Fu, Olson *et al*. *Proc Natl Acad Sci U S A* 1995, 92(6): 2333-2337.)
(TIF)

Table S1 **Primer sequences used in this study.**
(DOC)

Table S2 **H_2_ evolution in rice seedlings under light and dark conditions.**
(DOC)

Table S3 **H_2_ evolution of rice seedlings under the treatment of plant hormones and stresses (Data were shown as the concentration of H_2_ of the 2h measurement at every time points, ppm).**
(DOC)
