# Supplementary figures and images for: Molecular Hydrogen Is Involved in Phytohormone Signaling and Stress Responses in Plants
Source: PLoS One. 2013 Aug 12;8(8):e71038. doi: 10.1371/journal.pone.0071038 (PMC3741361; doi:10.1371/journal.pone.0071038)

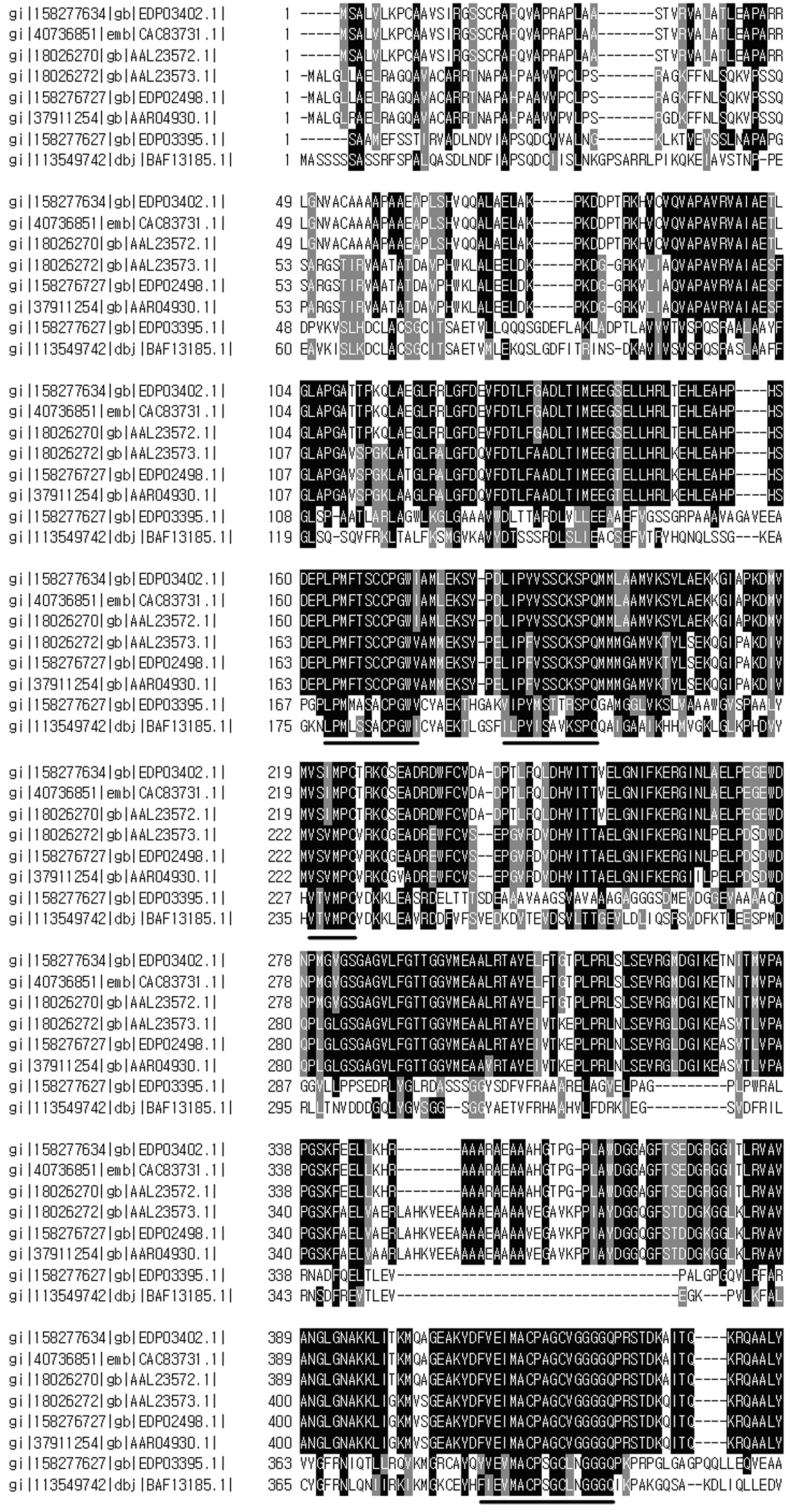

Supplement: Figure S1 — Amino acid alignment of predicted rice hydrogenase HydA1 protein (Accession No. BAF13185.1) and Chlamydomonas reinhardtii iron hydrogenase HydA1 proteins. Multiple sequence alignment was performed by ClustalX 1.81. Identical and similar residues are shaded in black and grey, respectively, by ISREC BOXSHADE software (http://www.ch.embnet.org/software/BOX_form.html). Conserved amino acids domains were underlined. Dashes (–) show gaps. (TIF) [file pone.0071038.s001.tif]

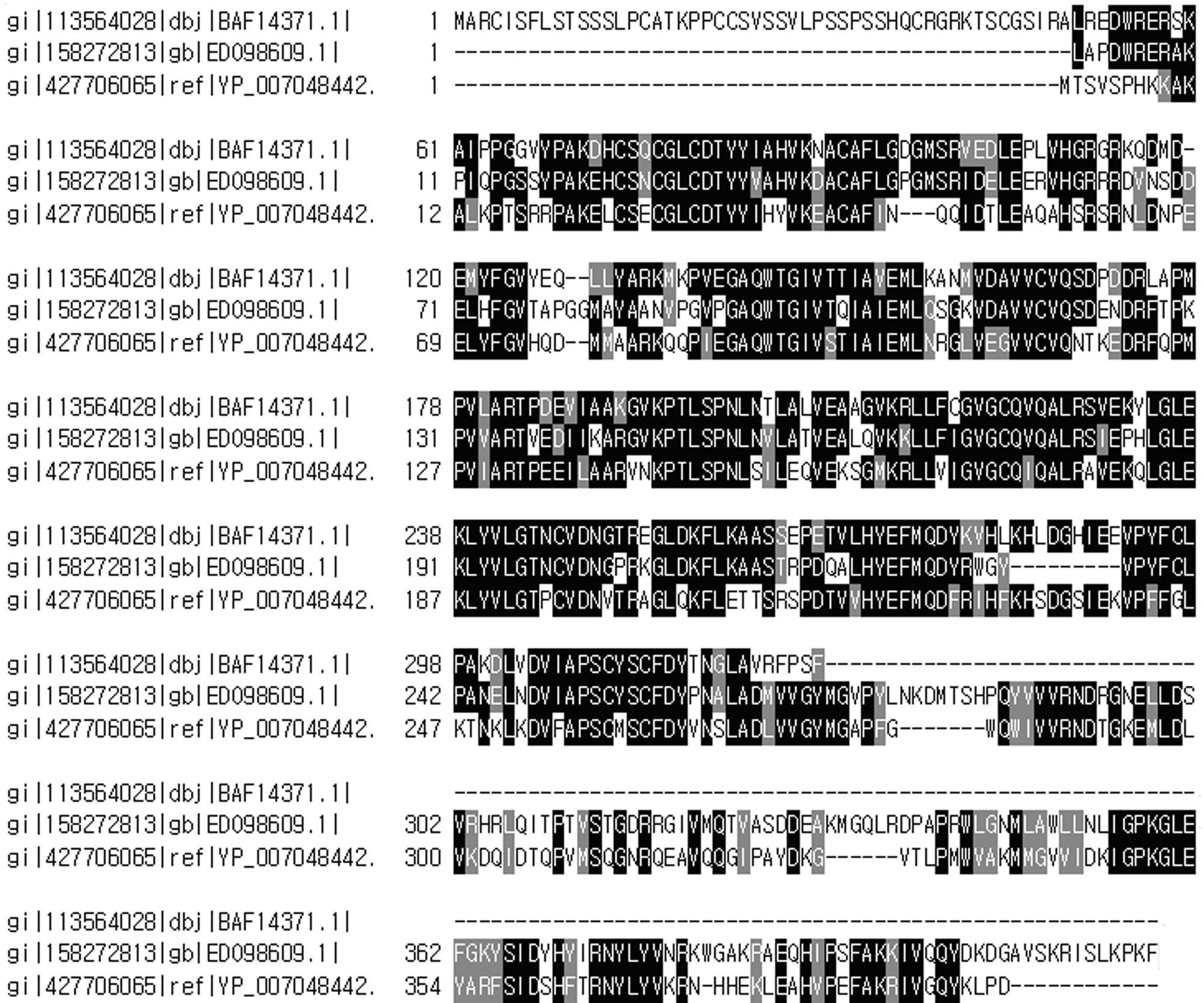

Supplement: Figure S2 — Amino acid alignment of predicted rice hydrogenase FhdB protein (Accession No. BAF14371.1) and other hydrogenase FhdB proteins (Nostoc sp. PCC 7107 (Accession No. YP_007048442) and Chlamydomonas reinhardtii (Accession No. EDO98609.1)). Multiple sequence alignment was performed by ClustalX 1.81. Identical and similar residues are shaded in black and grey, respectively, by ISREC BOXSHADE software (http://www.ch.embnet.org/software/BOX_form.html). Dashes (–) show gaps. (TIF) [file pone.0071038.s002.tif]

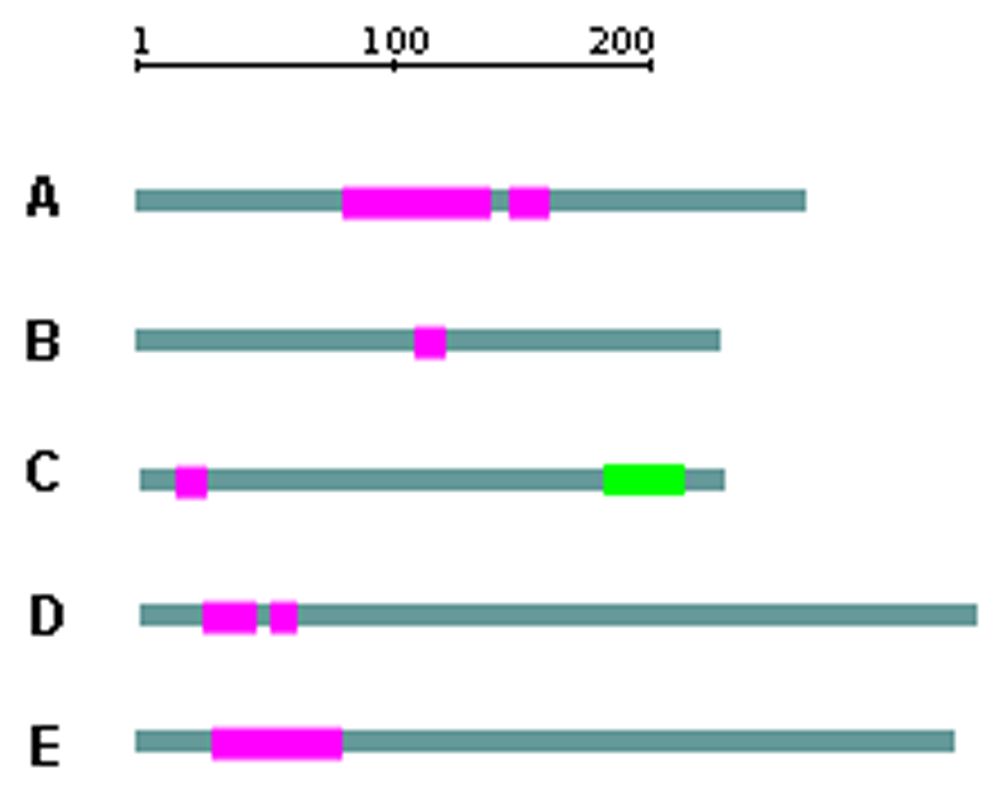

Supplement: Figure S3 — Domain analysis of predicted rice hydrogenase HypB protein (Accession No. BAF04766.1) (A) and other hydrogenase HypB proteins ( Methanothermobacter thermautotrophicus str. Delta H (Accession No. NP_275923.1) (B), Methanocella paludicola SANAE (Accession No. YP_003357614.1) (C), Cyanothece sp. PCC 7822 (Accession No. YP_003887299.1) (D) and Sphingopyxis alaskensis RB2256 (Accession No. YP_611146.1) (E). The domain analysis was performed at the website of SMART (http://smart.embl-heidelberg.de/), showing that HypB proteins have segments of low compositional complexity (in the color of pink). OsHypB has a histidine-rich region. The histidine residues within the histidine-rich region of HypB are involved in metal binding (Fu, Olson et al. Proc Natl Acad Sci U S A 1995, 92(6): 2333-2337.) (TIF) [file pone.0071038.s003.tif]
