# Supplementary material for: Molecular Hydrogen Is Involved in Phytohormone Signaling and Stress Responses in Plants
Source: PLoS One. 2013 Aug 12;8(8):e71038. doi: 10.1371/journal.pone.0071038 (PMC3741361; doi:10.1371/journal.pone.0071038)
Supplement: Table S1 — Primer sequences used in this study. (DOC) [file pone.0071038.s004.doc]

**Table S1.** Primer sequences used in this study

| **Name** | **Sequence (5' - 3')** | **GenBank Accession or LOC_ID** |
| --- | --- | --- |
| OsActin1 q-F | CGGTGTCATGGTCGGAAT | LOC_Os03g50885 |
| OsActin1 q-R | GCTCGTTGTAGAAGGTGT |
| OsERS1 q-F | TCATGGTTCTGATGCTTCCA | AF013979 (Note: ETH receptor) |
| OsERS1 q-R | TGCTCCATTAGCAGATCACG |
| OsERS2 q-F | CTCCCTCCAGACAGTGCAAG | AF460181 (Note: ETH receptor) |
| OsERS2 q-R | CATTACGGGCACAAATAGCC |
| OsETR2 q-F | GTTCGTCATCCAGTCGGAGA | AY136816 (Note: ETH receptor) |
| OsETR2 q-R | GAACTGAAGGGCAAGCATGA |
| OsETR3 q-F | GGATTTTCCATGTCCTGCTC | AY434735 (Note: ETH receptor) |
| OsETR3 q-R | TTCACCGAGGAGTAGCCAGT |
| OsETR4 q-F | GAATTGCCTCGTCGTTCTG | AY434734 (Note: ETH receptor) |
| OsETR4 q-R | GTTCAAGTGAGGGATGTACGG |
| OsACS1 q-F | GAGATGGTGAGCCAAGTGGT | AK071011 (LOC_Os03g51740) (Note: ACC synthetase) |
| OsACS1 q-R | CACTCCTCAAGCAGGTCGAA |
| OsAFB2-1 q-F | GAGCGGGATGGTAGCAATGAAATG | AK072338 (LOC_Os04g32460) (Note: Auxin receptor) |
| OsAFB2-1 q-R | CCGAGATAAGGGAGGCACACCAAC |
| OsAFB2-2 q-F | AAATGAGCGGGATGGTAGCAATGAAATG | AK100862 (LOC_Os04g32460) (Note: Auxin receptor) |
| OsAFB2-2 q-R | TAATGTATTGAAAGGGGTTACCTTATGG |
| OsTIR1 q-F | TCCAGGTGCTCCGCCTCGTCTCCT | EU400583 (LOC_Os05g05800) (Note: Auxin receptor) |
| OsTIR1 q-R | CCGGGAAGAGGCTGAGCCAATGAA |
| OsNPR4 q-F | CAACGTCGAGCAAATGTACG | AK067198 (Note: SA receptor) |
| OsNPR4 q-R | TCAAGCACTGGAGTCAGCTC |
| OsPR1 q-F | GGAAGTACGGCGAGAACATC | AF306651 (Note: Pathogen-related protein 1) |
| OsPR1 q-R | AGTTGCTCCACACCACCTG |
| OsGID1 q-F | GAGGCTGTTGGGTGGGTA | AB211399 (Note: GA receptor) |
| OsGID1 q-R | CCGCCGCAGAATGTTGTA |
| OsSLR1 q-F | ATCCCAACCTATCCCAAAGC | AB262980 (Note: SLR1 gene for DELLA protein) |
| OsSLR1 q-R | CGCCATCACCTTGTCCTT |
| OsPYL q-F | CACATCAAGGCTCCTGTTCA | AK065280 (Note: Orthologue of the ABA receptor**)** |
| OsPYL q-R | CTCTCCGTGCTGGTGGTC |
| OsHk6 q-F | CATGGACCGCGCCGAGG | BAD16039 (LOC_Os02g50480) (Note: CTK receptor) |
| OsHk6 q-R | GCCTGTCGCTTCATCGTC |
| OsHypB q-F | GCTGCCTAGCAACAAAGGTC | AK110758 (LOC_Os01g20830) (Note: Similar to Hydrogenase expression/formation protein hypB.) |
| OsHypB q-R | TACACCTCGCTCACACCATC |
| OsHydA1 q-F | ATTGCTCTCTCGTTCCGTTC | AK067853 (LOC_Os03g53750) (Note: Iron hydrogenase domain containing protein) |
| OsHydA1 q-R | AGGATTTGTGCTCACTGCAA |
| OsFhdB q-F | ACGAGTTGCGGGTCAATTAG | AK068015 (LOC_Os04g25400) (Note: Coenzyme F420 hydrogenase/dehydrogenase beta subunit, N-terminal domain containing protein) |
| OsFhdB q-R | CATCCATGTCTTGCTTCCTG |
| OsFeSOD q-F | TTGGATCTCTCCCAGTGAACA | AK062073 (LOC_Os06g05110) |
| OsFeSOD q-R | AGCCAGACGAACAACACCAA |
| OsMnSOD q-F | CGTCGCCAACTACAACAAGG | AK070528 (LOC_Os05g25850) |
| OsMnSOD q-R | CAAGTTTTGCATGTGGTGGA |
| OsCu/Zn SOD q-F | TTGGATCTCTCCCAGTGAACA | AK120348 (LOC_Os04g48410) |
| OsCu/Zn SOD q-R | AGCCAGACGAACAACACCAA |
| OsCAT-A q-F | GCAGAAACAAACCCCTCTCA | AK065094 (LOC_Os02g02400) |
| OsCAT-A q-R | TCTCGATCAGGTGGTAGTCCTC |
| OsCAT-B q-F | TCTCTTCTTCCCCACATCCA | AK100019 (LOC_Os06g51150) |
| OsCAT-B q-R | GTGAGGGCGGAGTTGTTGTT |
| OsAPX q-F | AAGAAGAAGCAGGGGAGCAT | AK061715 (LOC_Os07g49400) |
| OsAPX q-R | ATCGAAGGTGCCAGCAGAGT |
| OsGPX q-F | CCATCGTTCGTCCTCGTCT | AK062772 (LOC_ Os04g46960) |
| OsGPX q-R | CCTTCCCCTTGTAGGTGCTC |
