# Supplementary material for: Molecular Hydrogen Is Involved in Phytohormone Signaling and Stress Responses in Plants
Source: PLoS One. 2013 Aug 12;8(8):e71038. doi: 10.1371/journal.pone.0071038 (PMC3741361; doi:10.1371/journal.pone.0071038)
Supplement: Table S2 — H2 evolution in rice seedlings under light and dark conditions. (DOC) [file pone.0071038.s005.doc]

**Table S2. H_2_ evolution in rice seedlings under light and dark conditions**

| ***Seedling Number*** | ***H_2_ concentration in 250 ml glass beaker(ppm)*** | | | | | | ***Fresh Weight(g)*** | Evolution Rate (μmol gFW^-1^ h^-1^) | |
| --- | --- | --- | --- | --- | --- | --- | --- | --- | --- |
|  | 14:00pm | 16:00pm | 18:00pm | 20:00pm | 22:00pm | 24:00pm |  | **Light** | **Dark** |
| 1 | 7.76 | 8.26 | 7.98 | 7.66 | 7.82 | 7.50 | 1.7 | 0.294 | 0.281 |
| 2 | 11.86 | 12.08 | 12.18 | 11.57 | 12.02 | 11.78 | 2.5 | 0.301 | 0.293 |
| 3 | 14.93 | 14.32 | 15.16 | 14.73 | 15.17 | 14.32 | 2.8 | 0.330 | 0.328 |
| 4 | 21.24 | 18.85 | 19.88 | 17.68 | 20.05 | 18.32 | 3.6 | 0.347 | 0.318 |
| Average |  |  |  |  |  |  |  | **0.318** | **0.305** |
