# Supplementary material for: Molecular Hydrogen Is Involved in Phytohormone Signaling and Stress Responses in Plants
Source: PLoS One. 2013 Aug 12;8(8):e71038. doi: 10.1371/journal.pone.0071038 (PMC3741361; doi:10.1371/journal.pone.0071038)
Supplement: Table S3 — H2 evolution of rice seedlings under the treatment of plant hormones and stresses (Data were shown as the concentration of H2 of the 2h measurement at every time points, ppm). (DOC) [file pone.0071038.s006.doc]

**Table S3. H_2_ evolution of rice seedlings under the treatment of plant hormones and stresses (Data were shown as the concentration of H_2_ of the 2h measurement at every time points, ppm)**

| ***Seedling Number*** | ***Before Treatment*** | | | ***Treatment*** | After treatment | | | |
| --- | --- | --- | --- | --- | --- | --- | --- | --- |
|  | 20:00pm | 22:00pm | 24:00pm |  | 8:00am | 20:00pm | 12:00am | 24:00pm |
| 1 | 6.02 | 5.90 | 5.94 | **ABA**(1μg/ml) | 6.95 | 6.97 | 7.91 | 6.95 |
| 2 | 10.96 | 11.24 | 11.10 | **ABA**(1μg/ml) | 13.03 | 12.94 | 14.6 | 13.77 |
| 3 | 17.15 | 16.27 | 16.38 | **ABA**(1μg/ml) | 19.35 | 19.78 | 22.16 | 19.47 |
| 4 | 3.97 | 4.28 | 4.20 | **JA**(1μg/ml) | 8.62 | 5.83 | 3.95 | 3.28 |
| 5 | 16.86 | 17.30 | 17.72 | **JA**(1μg/ml) | 35.52 | 23.96 | 16.37 | 13.53 |
| 6 | 9.05 | 8.94 | 8.72 | **JA**(1μg/ml) | 18.23 | 12.35 | 8.38 | 6.92 |
| 7 | 5.60 | 5.48 | 5.55 | **NaCl**(1%) | 8.48 | 11.3 | 11.14 | 10.04 |
| 8 | 11.85 | 12.03 | 11.95 | **NaCl**(1%) | 18.43 | 24.42 | 25.78 | 21.75 |
| 9 | 8.47 | 8.30 | 8.38 | **NaCl**(1%) | 12.10 | 16.94 | 17.87 | 15.72 |
| 10 | 5.42 | 5.38 | 5.31 | **PEG6000**(5%) | 5.89 | 5.19 | 7.53 | 4.54 |
| 11 | 9.40 | 9.52 | 9.48 | **PEG6000**(5%) | 10.52 | 10.34 | 13.43 | 8.75 |
| 12 | 13.85 | 13.80 | 13.78 | **PEG6000**(5%) | 15.30 | 15.02 | 19.56 | 11.80 |
| 13 | 6.95 | 6.83 | 6.86 | **ETH**(1μg/ml) | 10.82 | 8.98 | 7.18 | 5.84 |
| 14 | 18.07 | 18.51 | 18.50 | **ETH**(1μg/ml) | 29.07 | 23.12 | 20.95 | 15.43 |
| 15 | 11.35 | 11.22 | 11.30 | **ETH**(1μg/ml) | 17.82 | 14.79 | 12.7 | 9.61 |
| 16 | 2.53 | 2.60 | 2.65 | **KT**(1μg/ml) | 1.3 | 2.37 | 2.27 | 2.46 |
| 17 | 5.52 | 5.41 | 5.36 | **KT**(1μg/ml) | 2.64 | 4.91 | 4.12 | 5.04 |
| 18 | 12.56 | 12.67 | 12.59 | **KT**(1μg/ml) | 6.46 | 11.79 | 11.04 | 11.83 |
| 19 | 11.78 | 11.88 | 11.84 | **GA_3_**(1μg/ml) | 7.44 | 7.29 | 6.41 | 7.34 |
| 20 | 6.53 | 6.95 | 6.23 | **GA_3_**(1μg/ml) | 4.15 | 4.04 | 3.62 | 3.94 |
| 21 | 14.71 | 14.67 | 14.58 | **GA_3_**(1μg/ml) | 9.27 | 9.16 | 8.07 | 9.78 |
| 22 | 10.92 | 11.00 | 10.97 | **IAA**(1μg/ml) | 6.69 | 5.51 | 4.81 | 5.55 |
| 23 | 7.87 | 7.92 | 7.63 | **IAA**(1μg/ml) | 4.18 | 3.99 | 3.41 | 3.61 |
| **24** | 14.93 | 15.05 | 15.16 | **IAA**(1μg/ml) | 9.15 | 7.62 | 6.62 | 7.64 |
